# A Field Study Method as a Potential Higher Tier Option to Refine Herbicide Risk Assessment for Nontarget Terrestrial Plants

**DOI:** 10.1002/ieam.4263

**Published:** 2020-04-16

**Authors:** Rena Isemer, Christine Mihan, Stephanie Peeters, Quintana Rumohr, Andreas Toschki, Virginie Ducrot

**Affiliations:** ^1^ Bayer AG, Crop Science Division, Monheim Germany; ^2^ gaiac Research Institute for Ecosystem Analysis and Assessment Aachen Germany

**Keywords:** Nontarget terrestrial plants, Higher tier, Field testing, Herbicide, Risk assessment

## Abstract

During herbicide spray application, nontarget terrestrial plants (NTTPs) growing in the off‐field area need to be protected from unacceptable effects of herbicide drift. The risk of such unintended effects is assessed in order to establish whether a particular use can be approved, possibly in combination with mitigation measures. In Europe, the risk of herbicide treatment to NTTPs is assessed on the basis of tier 2 studies done under controlled conditions in greenhouses. Following the concept of a tiered testing approach, higher tier field studies under more realistic conditions could be used to refine the risk assessment. No current guideline for conducting higher tier NTTP field studies is available. We developed an NTTP higher tier field study method done on an experimental plant community established by sowing of a seed mixture. The setup was optimized in 3 pilot field studies and subsequently used for a definitive study testing effects of the herbicide iofensulfuron‐sodium. Results show that the method can be regarded as a suitable higher tier option for assessing effects of herbicides on NTTPs. Growth of species from the soil seed bank cannot be avoided and has to be carefully considered when evaluating results. Adaptations of the study design may be necessary when testing different herbicides. Community‐level endpoints were at the same level as single‐species endpoints. Results of the field study were compared to standard greenhouse study results for the same herbicide. No observed effect rates (NOERs) in the field were about a factor of 10 higher and show that the current tier 2 risk assessment for NTTPs can be regarded as protective in this case. Whether the present field study design and the assessed endpoints can be used in higher tier risk assessment of NTTPs depends on selection of the specific protection goal and requires further discussion. *Integr Environ Assess Manag* 2020;16:691–705. © 2020 Bayer AG. *Integrated Environmental Assessment and Management* published by Wiley Periodicals, Inc. on behalf of Society of Environmental Toxicology & Chemistry (SETAC)

## KEY POINTS

- A method for testing effects of herbicides on nontarget terrestrial plants (NTTPs) in the field based on establishing an experimental plant community via sowing of a seed mixture of noncrop plant species on a test area was developed, optimized, and evaluated.
- The field method was used to assess effects of the herbicide iofensulfuron‐sodium on single‐species and community‐level endpoints based on vegetation coverage, emergence, phytotoxicity, growth stage, and total plant biomass, which showed that community endpoints cover single‐species endpoints.
- Field study and standard NTTP single‐species greenhouse study results were compared, and endpoints in the field were higher than in the greenhouse study.
- Representing a higher tier option, the proposed method increases realism alongside with variability of results and is, depending on the specific protection goal, potentially suitable to inform the risk assessment and serve as a reference tier.

## INTRODUCTION

Spray application of herbicides to crop production areas is a ubiquitous method to avoid competition for light, nutrients, and space by weeds; to minimize difficulties during harvesting; and to subsequently maximize crop yield. A certain fraction of the herbicide in the form of spray drift may reach nontarget terrestrial plants (NTTPs) growing in the off‐crop area (Boutin and Rogers 2000; EFSA 2014; Arts et al. 2015). This puts NTTPs at a risk of unintentional damage by herbicides. As important components of the ecosystem, they need to be protected from these unintended effects (EU 2009; EFSA 2014). In order to select appropriate protection measures (e.g., unsprayed buffer zones or drift‐reducing nozzles during application), the risk of herbicide use toward NTTPs needs to be assessed. Currently, the standard herbicide risk assessment for NTTPs in Europe is based on dose–response tier 2 greenhouse tests assessing effects of herbicide formulations on a minimum of 6 (mostly crop) species used as surrogates for NTTPs (OECD 2006a [test 208], 2006b [test 227]). In these tests, shoot dry weight, emergence, survival, shoot length, growth stage, and symptoms of phytotoxicity are the most frequently assessed endpoints. An ER50 (rate at which a test parameter is decreased by 50% compared to the control) is usually estimated. The ER50 values are used in combination with drift exposure values to inform the risk assessment. As a first conservative step, a deterministic risk assessment is done by directly comparing effects represented by the overall lowest ER50 to predicted exposure values to determine the so‐called “toxicity to exposure value” ([TER]: ER50 divided by estimated drift; EFSA 2002). According to current European Union (EU) regulations, a trigger value of 5 is used to guarantee protectiveness of the TER. In most cases, a probabilistic refinement step is added, whereby all suitable ER50 values are used in a species sensitivity distribution (SSD) (Posthuma et al. 2001; EFSA 2002) to calculate the HR5 (rate below which less than 5% of the species will be harmed above the ER50 level). By default, no assessment factor is used for the probabilistic approach because it is regarded to be protective in itself (EFSA 2002).

In order to guarantee a high reproducibility and low variability of the test results, the standard tier 2 NTTP studies are conducted mostly in greenhouses with crop species under controlled conditions, which are not necessarily representative of field conditions. This testing regime follows the concept of a tiered testing approach whereby lower tier tests are designed to ensure a high protection level and are conservative. For example in the standard tests, a few plant individuals grown as monocultures in single pots are assessed (OECD 2006a, 2006b). As only minor interception by other plants during application occurs (if at all), plants can be much more exposed to the herbicide under these conditions than if they were growing in a realistic plant community. This can lead to higher effects on some species in the greenhouse than under real world conditions (Marrs and Frost 1997; Brain et al. 2017, 2019). The cultivation as monocultures also prevents the assessment of possible community effects such as interspecies competition. In addition, test plants in the greenhouse are bottom watered in order to avoid wash‐off of the applied herbicide. This may increase exposure of the test plants to the herbicide beyond a realistic level and consequently lead to a very conservative view of the risk based on the greenhouse test. As it is the concept of a tiered testing approach, higher tier tests could be used to refine the risk assessment under increasingly realistic conditions. Field studies have been suggested as one higher tier test option, and several methods have been proposed (Marrs et al. 1991; Marrs and Frost 1997; De Jong and de Haes 2001; EFSA 2014; Brain et al. 2017, 2019; Nelemans et al. 2017; Strandberg et al. 2019). Nevertheless, no European standard guideline for higher tier NTTP field studies is available.

In the present paper, we present an NTTP higher tier field study method that allows for both comparing field and greenhouse sensitivity of NTTPs as well as incorporating the effects of potential interactions between species. We established experimental plant communities on a test field by sowing a seed mixture. The advantages of the experimental plant community (see also Nelemans et al. 2017) are the known composition of plant species, the fast and cost‐efficient population establishment (especially when compared to testing on preset meadows, etc.), good reproducibility in different regions, and a greater realism than some other proposed higher tier options such as using greenhouse‐grown plants for outside testing (Brain et al. 2017, 2019). The following 4 criteria were carefully considered for the development of a higher tier field study: 1) high reproducibility, 2) low variability, and 3) sufficient statistical power to detect small effects as well as 4) an overall good feasibility of the method. In 3 pilot field studies, the proposed study setup was evaluated for these 4 criteria and optimized accordingly. The optimized study design was used for a definitive study evaluating the effects of the sulfonyl‐urea herbicide iofensulfuron‐sodium applied by overspray. Results of the definitive field study were compared to greenhouse standard study results done with the same herbicide formulation. Because a field study represents a possible higher tier option or even a reference tier (EFSA 2014), the present paper intends to add research‐based information to the ongoing discussion on how to integrate field study results into the risk assessment for nontarget terrestrial plants and whether these results are really necessary to improve the current standard NTTP risk assessment scheme.

## MATERIALS AND METHODS

### Field studies

#### Pilot field studies

Three pilot field studies were conducted during the growing seasons of 2014, 2015, and 2016. Three different test sites (one in 2014, two in 2015, three in 2016) in western Germany were used to assess the effects of differing environmental conditions on the suitability of the test method. Experimental plant communities were established on test fields as a basis for conducting the field study by sowing seed mixtures. Because one objective of the pilot studies was to find a suitable seed mixture, several mixtures were compared. Among the tested seed mixtures were commercially available preset mixtures of 10 to 15 wild, gardening, and/or crop plant species (mostly seed mixtures intended for bee feeding or revegetation); self‐made mixtures of 11 wild, gardening, and crop plant species selected for expected good growth on the test fields and their sensitivity toward herbicides; and 1 mixture with 22 indigenous wild plant species typical for the growing region. Because the pilot studies were designed for optimization of the method, no herbicide was applied. Different cultivation and assessment methods as well as timelines for the studies were compared. The findings and experience from the 3 pilot field studies were used in 2017–2018 for a definitive field study, including application of a herbicide. Methods used in the present definitive study are described in the following sections.

#### Location and weather conditions

**Figure 1:**
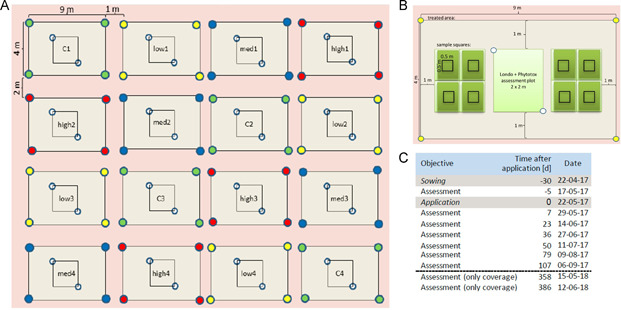
Study design and timing of assessments of the definitive field study in 2017. Schematic allocation of treatment areas (each 9 × 4 m) on the test field (**A**). Treatment with 3 herbicide rates (“low”, “med” = medium, and “high”) and a water control (**C**) was done at 4 treatment areas each (1–4). Treatments were evenly distributed on the test plot. Scheme of a treatment area (**B**). A 2 × 2–m assessment plot for assessment of vegetation coverage according to the Londo scale and assessment of phytotoxicity as well as eight 0.5 × 0.5–m sample squares were established. Time schedule of the field study in 2017–2018 (**C**).

In 2017, the study site was located in Höfchen, North Rhine‐Westphalia, on a field of 42 × 25 m. The soil was a loamy soil (1.9% organic matter; 16 mg/100 g soil P_2_O_2_, 18 mg/100 g soil K_2_O; 8 mg/100 g soil MgO; pH 6.5) and was not fertilized before or during the study. Before sowing on 22 April, the field was ploughed and tilled with a rotary harrow in order to reduce growth of the species of the soil seed bank. After the last assessment in 2017 on 6 September, the field was left undisturbed until the assessments in 2018 were finished (Figure 1C). Rainfall and temperature were recorded (Supplemental Data Figure 1).

#### Seed mixture

In order to establish an experimental plant community, a seed mixture was sown in April 2017 to the test field. The seed mixture “Blühweidenmischung MEKA 1” (MEKA mixture, “Bienenzuchtbedarf Seip”) was used in 2017 because it has proved to be the most appropriate for testing in the 3 pilot studies (e.g., good germination and homogeneous growth). The seed mixture comprises the following 11 annual, noncrop (“gardening”) species: *Borago officinalis*, *Calendula officinalis*, *Centaurea cyanus*, *Fagopyrum esculentum*, *Helianthus annuus*, *Linum usitatissimum*, *Malva* spp., *Papaver rhoeas*, *Phacelia tanacetifolia*, *Trifolium incarnatum*, and *Trifolium resupinatum*. To obtain a homogeneous distribution of seeds on the field and to facilitate the sowing procedure, the seed mixture was blended with semolina at a 1:4 ratio and then sown at 10 g seed+semolina mix per square meter.

#### Study design and herbicide treatment

Prior to herbicide treatment in 2017, the sown area was divided into 16 treatment areas (9 × 4 m each). Within each treatment area, a central assessment plot of 2 × 2 m was marked and surrounded by eight 0.5 × 0.5 m sampling squares, all marked by planting sticks of different colors (Figure 1A, B). Given that the test area had a slight slope with a moisture gradient, the different treatments were evenly distributed on the field (1 control and 1 treatment plot of each rate in each “line” and “column” of the test area, Figure 1A) to account for potential inhomogeneous growth of the sown plants. Three rates (low = 0.12 g active substance [a.s.]/ha, medium = 0.37 g/ha a.s., high = 1.10 g/ha a.s.) of a water dispersable (WG) formulation containing the sulfonyl‐urea herbicide iofensulfuron‐sodium (Wieczorek et al. 2017) and a water control were applied as direct overspray with a backpack sprayer with a volume rate of 300 L/ha using 4 DG110‐03 nozzles at 2 bar to 4 of the 16 treatment areas, respectively. The application rates were verified by checking the remaining volume in the sprayer after the application. The herbicide was applied in May (about 4 wk after sowing) when the majority of the plant species was in the 2 to 4 leaf stage, the same growth stage used in standard greenhouse testing for NTTPs.

#### Assessments

The plants' performance was assessed on 7 d in 2017 and on 2 d in 2018 (Figure 1C); first 3 wk after sowing (i.e., 1 wk before application of the herbicide) in order to determine an initial state. After application, the plots were assessed biweekly, except for assessments 6 and 7, which were done with 4‐wk intervals. This frequency was chosen according to findings from the pilot studies, which showed that during the earlier growth phase vegetation mapping is easier and more accurate due to lower plant density and a better homogeneity between replicates. The date of the application was regarded as day 0; all other dates are numbered correspondingly. Endpoints measured were vegetation coverage, overall vegetation height, symptoms of phytotoxicity, growth stage according to Biologische Bundesanstalt für Land‐ und Forstwirtschaft, Bundessortenamt und CHemische Industrie (BBCH), and shoot dry weight of total vegetation. The coverage of sown species and species emerging spontaneously from the soil seed bank was recorded visually using the Londo scale (Londo 1976). “Coverage” refers to the part of the soil surface that is covered by the plant species in vertical projection. The height of the vegetation on each assessment plot was estimated using a folding meter stick. Symptoms of phytotoxicity were assessed starting on day 7 for each sown plant species determining a median value for all plants in a plot. The following symptoms were examined in combination with a rating system from 0 to E used to determine the severity of the symptom: chlorosis (yellowing of green shoot tissue), necrosis (brown shoot tissue), bleaching (shoot tissue without pigmentation), leaf deformation (leaf curl, abnormal leaf shape), stunting (plant height reduced with shorter internode length), and any other symptom (e.g., reddening of plants). Rating system: 0 = no injury or effect, A = slight symptoms, B = moderate symptoms, C = severe symptoms, D = total‐plant symptoms, E = moribund. In addition, the growth stage of each sown plant species was assessed according to an extended BBCH scale (Hess et al. 1997). Fresh plant material was collected from the sampling squares allocated left and right of the assessment plots (Figure 1B) using a wooden frame (50 × 50 cm) and grass shears to cut the plants as close to the soil surface as possible. The plant material was stored in preweighted labeled bags and dried in a drying closet at 60 °C until the weight was constant. The 2 additional assessments in May and June 2018 were implemented to detect any potential long‐term effects, and only vegetation coverage (total and per species) was estimated.

### Greenhouse study

A standard tier 2 study was performed in a greenhouse, evaluating the effects of the same sulfonyl‐urea herbicide used in the field study in 2017 on the vegetative vigor of 10 nontarget plant species. The study followed Organisation of Economic Co‐operation and Development (OECD 2006b) guideline 227, United States Environmental Protection Agency (USEPA 1996) guideline OPPTS 850.4250, as well as good laboratory practice (GLP). The following 10 crop species were tested: *Beta vulgaris*, *Brassica napus*, *Cucumis sativus*, *Glycine max*, *Helianthus annuus*, *Lycopersicon esculentum*, *Allium cepa*, *Lolium perenne*, *Sorghum sudanese*, and *Zea mays*. A silt loam soil was used (16.7% sand, 59.1% silt, 24.2% clay, 1.3% organic matter, pH 7.31) and fertilized with 2.4 g/L fertilizer (Blaukorn) prior to sowing. The temperature in the greenhouse was set to 23 ± 8 °C during the day and 18 ± 8 °C during the night. The relative humidity was 70 ± 30%, and the photoperiod was at least 16‐h light. Natural daylight was supplemented by artificial lighting to provide the required photoperiod. At light intensities >15 000 lux, the lamps turned off, and at light intensities >50 000 lux, the shading closed. The same WG formulation containing the sulfonyl‐urea herbicide iofensulfuron‐sodium that was used for the field study mixed with an adjuvant and a water control were sprayed onto the plants at the 2 to 4 leaf stage in a spray chamber (Schachtner Tracksprayer SprayLab SLGH 2500) at a volume rate of 200 L/ha. Each plant species was tested with 5 to 6 test item rates ranging between 0.0015 and 10 g/ha a.s. Thirty‐two individuals were tested per treatment group with 4 plants grown together in 1 pot representing 1 replicate. The exposure duration in the greenhouse was 21 d. Plant survival was recorded 7, 14, and 21 d after application. Shoot length and shoot dry weight were assessed 21 d after application at termination of the study.

### Data analysis

For coverage of sown species, shoot dry weight, and vegetation height measured during the field study in 2017, differences between treatments and controls were statistically tested by means of the multiple *t*‐test by Williams (1972) using the program Community Analysis V4.3.12 (Hommen et al. 1994). The coverage data were log‐transformed (*y*′ = ln(*ay* + 1)) before the analysis, in order to meet normality and homoscedasticity (homogeneity of variances) requirements. All pairwise tests were performed 1‐sided with α = 0.05. Additionally, the minimum detectable differences (MDDs) were calculated for selected endpoints in accordance to Brock et al. (2015) with the same software package. At the community level in the field study, diversity indices as well as principal response curves (PRCs) (Van den Brink and Ter Braak 1998, 1999) were used for analyses. The program Community Analysis V4.3.12 was used for the calculation of the diversity indices. The diversity of the experimental plant community was described using 3 different measures: 1) the number of species (taxa richness) per treatment was plotted against time, 2) the Shannon‐Weaver Index (Streit 1980), and 3) evenness were calculated. The Shannon‐Weaver Index is a frequently used diversity measure that is dependent on species richness and frequency distribution of the individuals of a species (Boyle et al. 1990) and is the larger the more species are found and the more homogeneously the individuals are distributed on the species. The coverage data of all species, sown and spontaneously grown, were included in the PRC. A redundancy analysis restricted to each sampling date gave information if the treatments showed significant differences to the control in community structure at this date. If this were the case (*p* ≤ 0.05), a principal component analysis was applied to the data of that sampling date. The resulting sample scores were used as inputs in a multiple *t*‐test by Williams (1971, 1972) in order to test the effects on the community level. The standard greenhouse study was statistically analyzed with the ToxRat Professional software (version 2.10; ToxRat 2003) and no observed effect rates (NOERs), ER50s, and MDDs are reported.

## RESULTS

### Pilot field studies for method development (2014–2016)

Considering experience from the 3 pilot studies (2014–2016), measures were developed that help to fulfill the 4 key criteria for higher tier field studies. The measures are described in Table 1 and it is indicated which of the key criteria are influenced. Because a homogeneously growing plant community that can be reliably reproduced is an important prerequisite for the proposed test method, the choice of an appropriate seed mixture for establishment of the experimental plant community was crucial. From the several options tested in the pilot studies, 1 seed mixture comprising 11 noncrop, annual, dicoteledonous plant species proved to be most suitable (Blühweidenmischung MEKA 1). This seed mixture showed a good germination of species, homogeneous growth, and was commercially available in a consistent quality. Additional measures developed in the 3 pilot studies were a suitable pretreatment of the test field followed by an appropriate seeding procedure, selection of measurable assessment endpoints, as well as the right timing for starting the field study (Table 1).

**Table 1:** Measures for successfully conducting an NTTP higher tier field study in the proposed design, ensuring high reproducibility, low variability, and high sensitivity of the test system as well as an overall good feasibility^a^

Measure	Description of measure	Ensured key criteria	Limitation
Pretreatment of test acre	Several rounds of tilling with 2‐wk intervals starting in February to reduce growth of species from the soil seed bank	R, V	Growth of species from the soil seed bank can be reduced but not avoided completely
	No use of fertilizer to avoid excessive overgrowth of a few species		
Seeding procedure	Ensure homogeneous distribution of seeds by seeding as mixture with semolina	R, V, F	Due to the competition by species from the soil seed bank, unfeasible weather conditions, or foraging wildlife not all species will germinate and grow homogeneously
	Waltz after seeding to increase germination		
Seed mixture	Chose commercially available seed mixture with appropriate number of annual noncrop species to ensure homogeneous growth of a representative plant community	R, V, S, F	Species from the soil seed bank outcompete some of the sown species
			Adaptation of seed mixture according to the herbicide under assessment (differing modes of action and sensitive species) may be needed; change of seed mixture would require new development of method
Assessment method/endpoints	Coverage of sown species, total plant coverage (according to Londo scale), vegetation height, BBCH stage, phytotoxic symptoms, and shoot dry weight of total vegetation proved to be most suitable	R, S, F	
	Use trained personelle		
Timing	Sowing in March/April will reduce growth of species from the soil seed bank	R, V, S, F	Weather conditions may lead to change in timing
	Application of herbicide to seedlings in the 2–4 leaf stage in May		Plant stage at application is crucial and may impact outcome of the study
	Biweekly assessments in first 2 mo after application; monthly assessments in following 2 mo		

BBCH = Biologische Bundesanstalt für Land‐ und Forstwirtschaft, Bundessortenamt und CHemische Industrie; F = good feasibility of the method; NTTP = nontarget terrestrial plants; R = high reproducibility; S = high sensitivity; V = low variability.

^a^ The proposed measures were developed in 3 consecutive pilot field studies and proved to be effective. Some limitations seem to be inevitable.

Nevertheless, several limitations of the proposed NTTP higher tier field study design could not be completely overcome by the implemented measures (Table 1). The most important limitation was the spontaneous emergence of plant species from the soil seed bank. It was not possible to completely suppress growth of these species in the proposed design because pretreatment with a herbicide, soil treatments (e.g., steaming), complete exchange of soil, or other methods that would destroy the soil seed bank are not feasible (some of these methods have been tested in the pilot studies). Another limitation is the inhomogeneous growth of plants in the experimental community. Even though the measures proposed here could not completely overcome the inevitable limitations, they were suitable to reduce them. Species from the soil seed bank like *Chenopodium album* or *Sinapis alba* made up large proportions of the total plant coverage in the first pilot study in 2014 (up to 50% for *Chenopodium album*, up to 18% for *Sinapis alba*). Although up to 38 spontaneous species were still detected in 2016, the optimized measures and improved study design reduced their abundance in most cases to only a few individuals per species per plot. Abundance of *Chenopodium album* was reduced to a maximum of 25% of total coverage and abundance of *Sinapis alba* to a maximum of 5%.

### Definitive field study (2017–2018)

In the definitive study in 2017–2018, short‐ and long‐term effects of the sulfonyl‐urea herbicide on growth of 11 sown species under field conditions were assessed. In addition to the assessments on single‐species level, assessments and data analysis addressed the effects of the herbicide on the whole experimental plant community (i.e., total plant coverage, shoot dry weight, plant height = community‐level endpoints). Given that the growth of species from the soil seed bank is unavoidable, these species were included in the community level assessments and analysis.

#### Species composition of the experimental community

**Figure 2:**
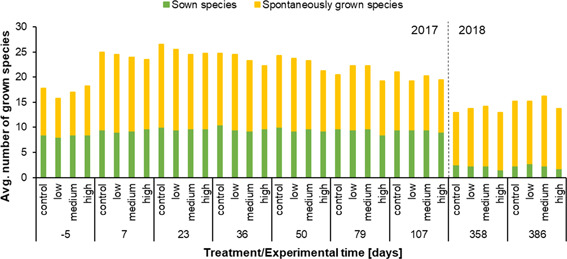
Average number of species on the control and treatment plots (*n* = 4 each) at the respective assessment days in 2017 and 2018. The average number of sown species is shown in green and of spontaneously grown species in yellow. The number of species was averaged over all 4 replicate plots.

Most of the 11 species in the sown mixture had already emerged at the first assessment day (Figure 2, day –5). Some species like *Calendula officinalis*, *Malva verticillata*, and *Papaver rhoeas*, were not present in every assessment plot. Hence, averaged over all plots, the number of sown species was below 10 one week before the application. A similar number of spontaneously grown species from the soil seed bank was recorded at the time. On day 7 (1 wk after the application) the total number of species had increased over all plots. Although the number of sown species was stable until day 50, the number of spontaneously grown species seemed to show a slight decrease with increasing treatment concentrations. Overall, no clear relationship could be observed linking the herbicide treatment and the number of the sown species. Two assessments in the following year 2018 (days 358 and 386) showed that although the abundance of spontaneously grown species was similar to the one in the previous year (days 7 to 36), only a few of the sown species managed to germinate and grow again in the following season. This was observed on all plots regardless of the treatment except for the high treatment plots where the number of sown species was slightly more reduced compared to the other plots assessed in 2018 (Figure 2).

#### Coverage, growth stage, and phytotoxicity assessment of sown species

**Figure 3:**
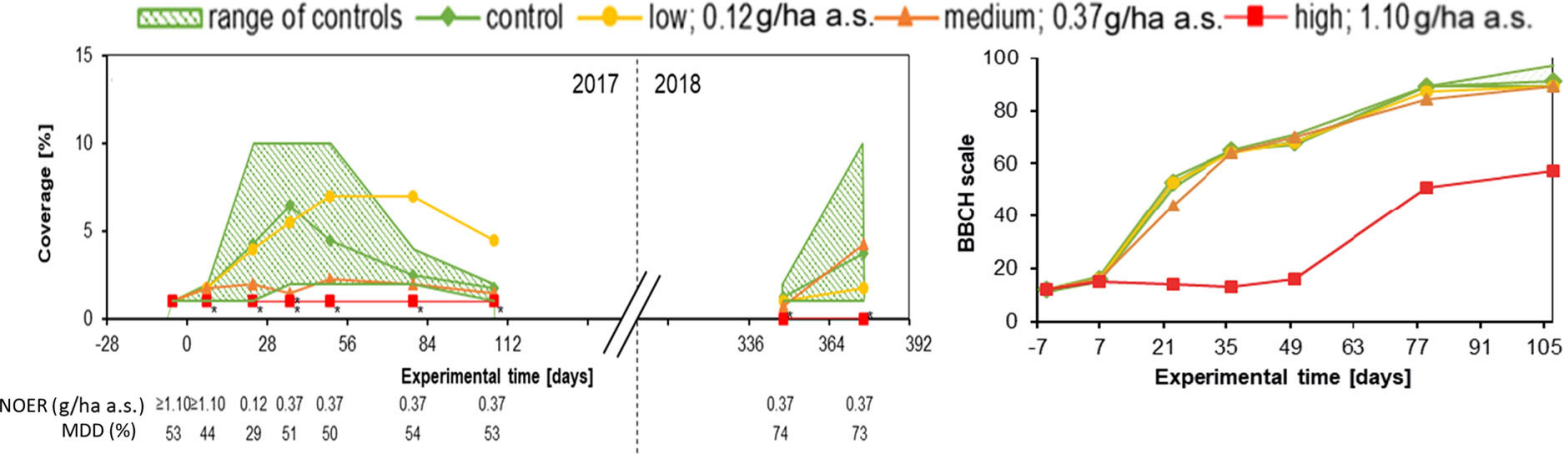
Vegetation coverage and development of *Borago officinalis* in 2017 and 2018. Average plant coverage with corresponding NOERs and MDDs for 2017 and 2018 (left) and BBCH growth stage for 2017 (right) for the control and the 3 treatment groups at the respective assessment days. * = significant differences according to Williams' *t*‐test with *p*‐value < 0.05; BBCH was not statistically evaluated. a.s. = active substance; BBCH = Biologische Bundesanstalt für Land‐ und Forstwirtschaft, Bundessortenamt und CHemische Industrie; MDD = minimum detectable difference; NOER = no observed effect rate.

The plant species of the seed mixture were assessed for growth inhibition, coverage, and phytotoxicity species by species. Most species were affected by the highest rate tested but showed no response at the low and medium rate (Figure 3; Supplemental Data Figures 2 and 3 and Supplemental Data Tables 1–11). Because it is expected under realistic but uncontrolled outdoor conditions, variability was high for some endpoints, for example, on treatment plots the coverage for *Borago officinalis* was within the range of the control (minimum to maximum measured values) at most assessments because control ranges were wide (Figure 3). For some species (e.g., *Malva* spp., *Calendula officinalis*) coverage was very low, so that no meaningful assessments were possible (Supplemental Data Figure 2). Negative but also positive effects of the herbicide treatment were observable at the single‐species level. For *Borago officinalis*, a clear negative effect on coverage and phytotoxic symptoms was observed at the high and medium treatment and also on the BBCH stage by the high treatment (Figure 3; Supplemental Data Table 1). The coverage in the low treatment plots was increased compared to the control, especially during later assessments in 2017. In 2018, *Borago officinalis* was detected on all but the high treatment plots with a similar coverage as in the previous year. The lack of *Borago officinalis* in the high treatment plots in 2018 could be related to its considerably delayed growth in 2017, which is likely to have affected seed production.

#### Vegetation height

Mean vegetation height was very similar between all treatments and no significant differences to the control were detected in 2017 (data not shown). This might have been due to the high variance of the 4 replicates of each treatment group. An exception was a significant increase in vegetation height of the high treatment plot on the first assessment day (358) of 2018.

#### Total plant coverage

**Figure 4:**
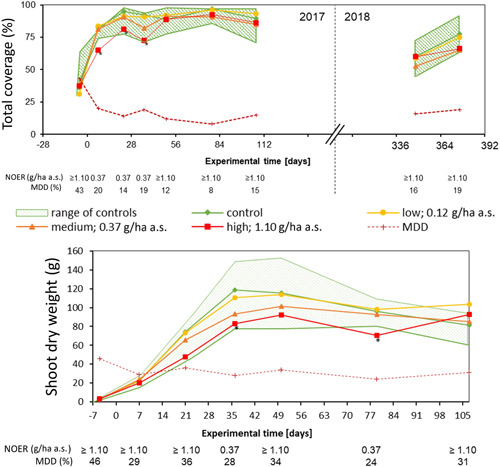
Total plant coverage (top) and total vegetation shoot dry weight (bottom) with corresponding NOERs and MDDs. * = significant difference according to Wiliams' *t*‐test with *p*‐value < 0.05; a.s. = active substance; MDD = minimum detectable difference; NOER = no observed effect rate.

In 2017, the total plant coverage was very similar between the treatment groups and the control group except for the highest treatment (Figure 4). Five days before treatment (day −5), all means of total coverage were in the range of variation of the control plots, showing homogenous growth of the experimental plant community. Homogeneity of the experimental plant community is an important prerequisite for detecting statistically relevant effects of the herbicide treatment. The coverage of the control plots increased further with the overall growth of vegetation in spring and remained stable until the end of the experiment in late summer. After application, the means of coverage of the low and medium treatment group stayed within the range of variation of the control group throughout the assessment period. The total coverage of the high treatment plots was decreased and differences to the control were statistically significant on the first 3 assessment days. From day 50 onward, the total coverage on the high treatment plots was similar to the coverage of the other plots. In 2018, the total coverage was similar between the treatments and the control, and the range of coverage at the beginning of the vegetation period was comparable to the previous year.

#### Shoot dry weight of total vegetation

At the first 2 assessment days (days −5 and 7), the total shoot dry weight was low (<20 g) due to the early time in the growth period (mid‐May). Because of the low dry weight, it was difficult to detect possible growth retardation effects of the herbicide used and to calculate reliable statistical differences (Figure 4). This is reflected by an MDD of 46%. For the parameter of shoot dry weight, this is the largest MDD calculated, given that MDDs for the other assessment dates ranged from 24% to 36% (Figure 4). Overall, MDDs were low for a field study and all fall within class IV (<50%, small effects can be determined) of the proposed European Food Safety Authority (EFSA) classification system for MDDs (EFSA 2013). From day 7 to day 50, the shoot dry weights of all treatment groups stayed within the range of the control group. Although the shoot dry weight of the low treatment group was near to the control average, the medium and high treatment groups showed a delayed increase. However, differences were statistically significant only for the high treatment group on day 36. At the last assessment day of the study in 2017, the measured total vegetation shoot dry weight of the treatment plots and the control were in the same range. For most of the assessments, even the highest treatment did not have a significant effect, and the NOER for shoot dry weight was associated with the highest treatment. Only on days 36 and 79 was the NOER at the level of the medium treatment, showing significant effects of the highest treatment on shoot dry weight.

#### Diversity analysis

**Figure 5:**
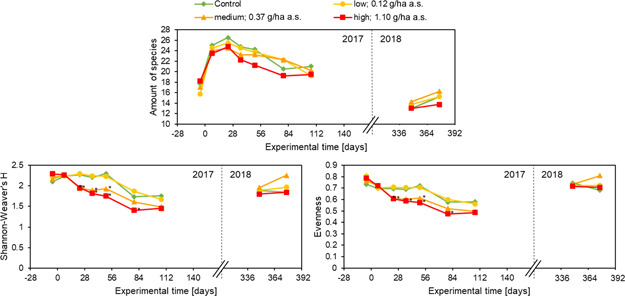
Diversity analysis: Amount/richness of species (top), Shannon‐Weaver Index (bottom left), and evenness (bottom right) were calculated for the species coverage data from 2017 and 2018. Mean values for the treatments and the control are shown. * = significant difference according to Wiliams' *t*‐test with *p*‐value < 0.05; a.s. = active substance.

At the first assessment day in 2017, many species were present only in a very juvenile stadium and therefore difficult to distinguish. Some species may not have germinated at all at that time. This resulted in a low number of species detected in the field. From the second assessment onward, the number of species (richness of species) increased in all plots for the next 4 wk (Figure 5). Although there was a slight decrease in the number of species visible as a result of the treatments, no statistically significant differences between the control and the different treatments were detected. In the next vegetation period in 2018, the richness of species was much lower for all treatments compared to the previous year. This observation could be due to the low germination of the species of the seed mixture sown in the previous year as well as to the absence of management of the field. This assumption is supported by a shift of the composition of spontaneously grown species, which showed an increase in grassy species and a decrease in crop accompanying species. Although there was no statistically significant difference between treatments and control for the species richness, evenness showed differences between the medium treatment and the control on days 23 and 50, and between the high treatment and the control on days 23 and 79 (Figure 5). Evenness of the low treatment was similar to the control throughout the study. The Shannon‐Weaver Index showed a similar trend to evenness, including statistically significant differences to the control for medium and high treatments on days 23 to 50 and days 23 to 79, respectively (Figure 5). No statistically significant differences between the treatment and control plots were found for evenness or the Shannon‐Weaver Index in 2018. In addition to the diversity analysis, similarity analysis (Steinhaus Index and Standers Index) likewise delivered no significant difference of the treatments compared to the control (data not shown).

#### Community analysis by PRCs based on species response data

**Figure 6:**
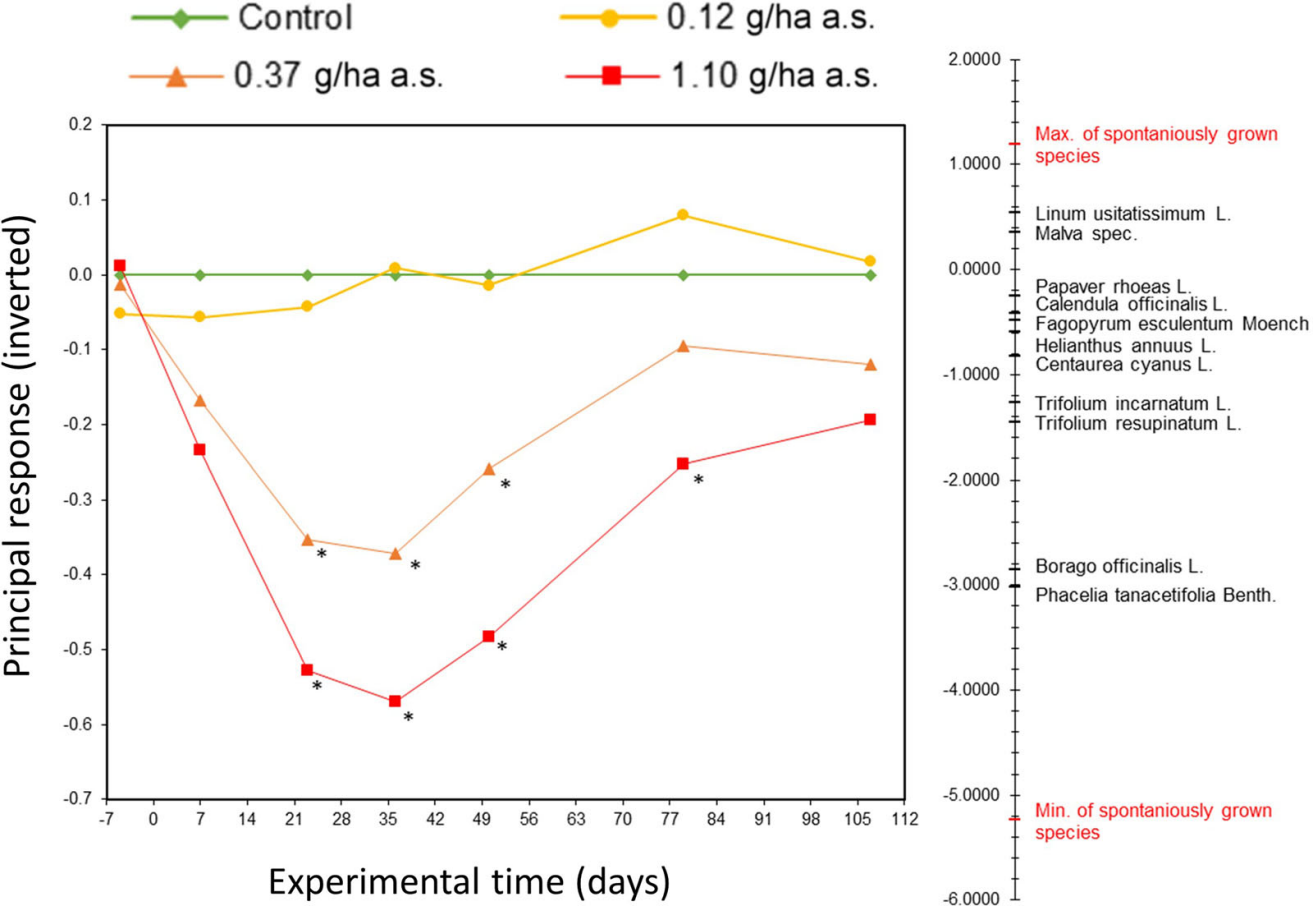
Inverted principal response curve (PRC, graph on the left) based on the species coverage data collected in 2017 and inverted species weights (right). The species weights are shown only for the sown species, and the minimum and maximum species weights of the spontaneously grown species from the soil seed bank are given. * = significant difference according to Wiliams' *t*‐test with *p*‐value < 0.05; a.s. = active substance.

Prior to herbicide application, all treatment groups were within a similar range as the control (Figure 6). After application, the low treatment group remained at a similar level as the control group and was never significantly different throughout the assessment period in 2017. The treatment scores of the medium and high treatment groups showed an immediate decrease after the application (day 7), resulting in a significant difference from the control from day 23 onward. The curve of the medium treatment group ascended toward the control level between day 36 and day 79, the latter assessment day showing no significant differences to the control. The high treatment group curve, which showed a stronger decrease than the medium treatment group, ascended again between day 36 and day 107, the latter date without significant differences to the control.

The (inverted) species weights for the sown taxa were evenly distributed over the range of species weights of all taxa (Figure 6). Only a few spontaneously grown taxa (7 out of 56) showed a higher species weight than taxa from the sown mixture. The second and third highest species weights belonged to *Phacelia tanacetifolia* and *Borago officinalis*. Most of the taxa, including *Centaurea cyanus*, *Helianthus annuus*, *Fagopyrum esculentum*, *Calendula officinalis*, and *Papaver rhoeas*, had species weights between −1.0 and 1.0 and thus were within the variance of the experimental community having only minor influences on the PRC.

### Comparison of field study results to standard greenhouse study results

In order to evaluate whether the current standard risk assessment for NTTPs based on greenhouse studies and ER50s is protective in the presented case (i.e., whether field endpoints are higher or lower than greenhouse endpoints), the field study results were compared to results of a standard NTTP greenhouse study. Plant studies in the field generally generate variable results. In a similarly designed field, where effects above the 50% level were observed, no statistically significant differences between control groups and treatments at the 50% effect level were detectable (Nelemans et al. 2017). Accordingly, the NOER and not the ER50, which is the standard effect value in the current regulatory context for NTTPs, would be the value to use for the risk assessment based on field data due to their intrinsic variability. Greenhouse studies, on the contrary, are designed to deliver ER50s (or other ER*x* values). No observed effect rates can be derived from the standard greenhouse tests as well, but they tend to be less statistically robust and sometimes can only be displayed as unbound (<) values. A direct comparison between greenhouse and field study results may accordingly be done based on comparing NOERs or comparing field NOERs with greenhouse ER50s (possibly even including the assessment factor of 5, which is currently used for the standard risk assessment).

The standard greenhouse vegetative vigor study for iofensulfuron‐sodium showed that *Beta vulgaris* (NOER shoot dry weight = 0.01 g/ha a.s.; ER50 shoot dry weight = 0.08 g/ha a.s.), *Glycine max* (NOER shoot length <0.04 g/ha a.s.; ER50 shoot length = 0.16 g/ha a.s.), and *Helianthus annuus* (NOER shoot dry weight <0.12 g/ha a.s.; ER50 shoot dry weight = 0.53 g/ha a.s.) were the most sensitive among the 10 tested crop species (Table 2). The overall lowest bound NOER and ER50, respectively, were determined for *Beta vulgaris*. The ER50 of 0.08 g/ha a.s. would accordingly be used to inform the standard NTTP risk assessment. In the field study, NOERs were determined for the various endpoints (plant coverage, shoot dry weight, etc.) on single‐species and community levels. The lowest NOER for the field study was 0.12 g/ha a.s. This NOER was the same on the single‐species level as well as on the community level.

**Table 2:** Comparison of field study NOERs to standard greenhouse study NOERs

Field study^a^		Greenhouse study	
Species (Family), measurement	Lowest NOER (g/ha a.s.)	Species (Family), measurement	Lowest NOER (ER50) (g/ha a.s.)
**Species endpoints** ^b^		**Species endpoints** ^b^	
*Borago officinalis* (Boraginaceae), coverage	0.12	*Beta vulgaris* (Amaranthaceae), shoot dry weight	0.01 (0.08)
*Calendula officinalis* (Asteraceae), coverage	≥1.10	*Brassica napus*, (Brassicaceae), shoot dry weight	0.12 (0.33)
*Centaurea cyanus* (Asteraceae), coverage	0.12	*Cucumis sativus* (Cucurbitaceae), shoot dry weight	0.37 (5.14)
*Fagopyrum esculentum* (Polygonaceae), coverage	≥1.10	*Glycine max* (Fabaceae), shoot length	<0.04 (0.16)
*Helianthus annuus* (Asteraceae), coverage	≥1.10	*Helianthus annuus* (Asteraceae), shoot dry weight	<0.12 (0.53)
*Linum usitatissimum* (Linaceae), coverage	n.c.	*Lycopersicon esculentum* (Solanaceae), shoot dry weight	1.10 (4.11)
*Malva* spec. (Malvaceae), coverage	≥1.10	*Allium cepa* (Amaryllidaceae), shoot dry weight	0.37 (1.29)
*Papaver rhoeas* (Papaveraceae), coverage	0.12	*Lolium perenne* (Poaceae), shoot dry weight	0.37 (0.91)
*Phacelia tanacetifolia* (Boraginaceae), coverage	0.12	*Sorghum sudanese* (Poaceae), shoot dry weight	0.37 (2.04)
*Trifolium incarnatum* (Fabaceae), coverage	0.12	*Zea mays* (Poaceae), shoot dry weight	1.10 (2.83)
*Trifolium resupinatum* (Fabaceae), coverage	0.12		
**Community endpoints** ^b^			
All species/total coverage	0.37		
All species/vegetation height	≥1.10		
All species/shoot dry weight	0.37		
All species/community coverage (PRC)	0.12		

a.s. = active substance; NOER = no observed effect rate; PRC = principal response curve.

^a^ Field study results are shown for species endpoints (single‐species coverage for the sown seed mixture) and community endpoints (total vegetation coverage, vegetation height, shoot dry weight, and community coverage [PRC]).

^b^ For each species measured in the 2 study types as well as for each community endpoint, the lowest NOER measured is shown. 0.12 g/ha a.s. = low treatment, 0.37 g/ha a.s. = medium treatment, 1.10 g/ha a.s. = high treatment in the field study; n.c.: not calculated due to positive effects.

In order to judge the statistical power of the 2 test systems, MDDs can be compared. For the greenhouse study, MDDs ranged from 5% to 36% (actually 5% to 23%, if *Sorghum sudanese* results would not be considered) (Supplemental Data Table 12). A comprehensive analysis of standardized greenhouse tests showed that MDDs typically range from 5% to 39.5% depending on test system and tested parameter (Staveley et al. 2018). The performed vegetative vigor study lies well within this range. For the field study, MDDs were less than 50% (class IV, small effects can be determined; EFSA 2013) for shoot dry weight and total plant coverage (Figure 4). For single‐species coverage, MDDs were variable depending on plant species and timepoint and ranged from 24% to 195% (Figure 3; Supplemental Data Figure 2).

## DISCUSSION

### Suitability of the method

To date, NTTP field studies have been performed based on a variety of methods, ranging from designs that are closer to the standard studies testing potted greenhouse‐grown plants planted as monocultures outside (Marrs et al. 1989; Brain et al. 2017, 2019) to realistic field studies on real field margin communities (Schmitz et al. 2014). The study design proposed here represents an intermediate approach. It adds an important increase in realism compared to the lower tier standard greenhouse studies: testing on a plant community instead of potted plants in monocultures and testing in the field in ambient conditions instead of in the controlled environment of a greenhouse. In addition, by testing on an experimental plant community instead of a natural community, the inevitable increase in variability and decrease in reproducibility that goes along with increased realism is kept as small as possible. To add an additional layer of realism, the use of wild plant species for establishing the experimental plant community instead of the used “gardening” species would be an option (compare discussion in EFSA 2014). Wild plant species have been tested for their suitability to establish a homogeneously growing plant community in a reasonable time frame in the pilot field studies (2014−2016) and were found to be unfit. Due to low germination, the sown wild species were outgrown by the soil seedbank species in most of the cases. Similar observations were made by Strandberg et al. (2019). Furthermore, a recent well‐performed literature review showed that wild plant species appear to be of a similar sensitivity toward herbicides like other plant species and the NTTP risk assessment based on tests performed with “nonwild” species is protective for all species (Christl, Morilla et al. 2019). The recommended MEKA 1 seed mixture comprises 11 species from 7 plant families. Most of these 11 species show a good germination and growth. Similar seed mixtures have already been used in similar study designs but with only few of the plant species showing a good emergence (Nelemans et al. 2017; Strandberg et al. 2019), which is probably due to the dominance of spontaneously growing species from the soil seed bank. The MEKA 1 seed mixture contains only annual dicotyledonous plants, which are suitable for testing a sulfonyl‐urea herbicide, because this class of herbicides is known to predominantly affect dicotyledonous plants. Given that other commercially available herbicides also specifically reduce dicots, the chosen seed mixture may be suitable for testing a variety of herbicides. Testing other classes of herbicides with different modes of action may require an adaptation of the used seed mixture, for example, by adding monocotyledonous species for testing herbicides that control grasses. Furthermore, using just annual plants allows the proposed study design to be applied within 1 growing season, whereas perennial plants would require a longer, unfeasible establishment time of the experimental plant community. It has been indicated that plants growing in arable areas are mostly annuals (Lososová et al. 2006). In addition, when choosing a seed mixture for testing, the climatic zone of the test must be considered. The seed mixture needs to consist of species that will grow under the regional climatic conditions. It has to be kept in mind that any change of seed mixture may require additional adaptations (timing of sowing and assessments, choice of growth area, and measured parameters) that will require additional time‐consuming optimizations of the study design, which might even require additional pilot studies. Also, timing of application needs to be carefully considered: Some species may not have emerged yet or may not be in the most vulnerable growth stage during applications. To determine the most realistic time of application and application scenario, label instructions of the tested herbicide could be considered.

Minimum detectable differences have been used to aid interpretation of results of field studies (EFSA 2013; Brock et al. 2015). The MDDs for the field study presented here fall mostly within class III or IV and indicate that the study is suitable for detecting small to medium effects. Nevertheless, the highest MDD is at 195%, reflecting high variability of measurements. Given that it is the case for most higher tier studies, the field study MDDs are higher than those of the standard greenhouse studies. A general remark on the suitability of the method for detecting effects based on MDDs should be made only after several studies with the same methodology have been done and assumptions can be based on a larger data set.

It has to be emphasized that several limitations of the proposed NTTP higher tier field study design could not be overcome by the implemented measures. It was not possible to completely suppress growth of the species emerging from the soil seed bank. This was also the case in other similar studies (Nelemans et al. 2017; Strandberg et al. 2019). Under certain environmental conditions, the seed bank species can become dominant and outcompete the sown species, thereby limiting the study's reproducibility and subsequently its usability for the risk assessment. In addition, the seed bank species tend to grow in patches on the test area and therefore increase variability between replicates as well as between trials. As these species are not prechosen to be sensitive to the tested herbicide (e.g., for the present case, they include some monocotyledonous species that are not sensitive to a sulfonyl‐urea herbicide), they also influence sensitivity and the statistical power of the test system. Overall, dominance of seed bank species may limit the usefulness of the study results for the risk assessment by adding a factor of uncertainty. To keep the field study results as standardized and comparable as possible, these species should not be included in the single‐species assessments, at least not when using the proposed study design. Because they are part of the plant community, they nevertheless need to be included in the community assessments. Another limitation is the overall inhomogeneous growth of plants in the experimental community. Homogeneity is heavily influenced by the growth of the soil seed bank species as well as environmental conditions such as soil quality and moisture gradients. It influences reproducibility, variability, and overall feasibility of the method. Lack of homogeneity may be manageable to a certain extent by increasing the number of replicates and/or choosing more homogeneous plots from a bigger experimental area. Both options nevertheless would increase the need for space, experimental labor, and subsequently costs for the study without guaranteeing improvements. They may lead to improving MDDs for the field study. These inevitable limitations of the study design are due to the more realistic nature of a field study and intrinsic variability of complex test systems, which need to be carefully considered when discussing the suitability of results to inform the risk assessment for NTTPs.

Testing effects of herbicides on reproduction of NTTPs during field studies to inform the risk assessment have been frequently demanded and investigated by researchers and authorities (e.g., Boutin et al. 2013; EFSA 2014; Schmitz et al. 2014; Nelemans et al. 2017; Strandberg et al. 2019). The presented field study design could be extended to measure reproductive parameters, as was done in other similar studies (Boutin et al. 2013; Nelemans et al. 2017; Strandberg et al. 2019). Flowering, seed production, and seed germination are discussed as possible assessment endpoints (Arts et al. 2017). Care must be taken when choosing the parameter: Flowering and seed production can be highly variable and therefore not suitable to deliver reliable endpoints. They are technically challenging to measure (experiences made by the authors during attempts to assess flowering in the pilot studies; data not shown). A recent literature review has revealed that a switch from vegetative to reproductive endpoints would increase conservatism of NTTP testing by only a factor of 1.5 (Christl, Hoen et al. 2019) which makes labor‐, time‐, and cost‐intense testing of reproduction questionable.

### Results and learnings from the definitive study

The results obtained in the definitive study can be discussed as single‐species endpoints or results (coverage, growth stage, and phytotoxic symptoms) and as community endpoints or results (total vegetation coverage and biomass, vegetation height, diversity analysis, PRC).

#### Single‐species endpoints

The plant species of the seed mixture showed different sensitivity toward the herbicide. Although most species showed effects at the medium or high test rates and accordingly had an NOER corresponding to the low test rate, some species like *Linum usitatissimum* seemed to benefit from the herbicide treatment: Because they emerged after the application they were not affected by the herbicide and benefited subsequently from less competition by those species that were directly affected. *Borago officinalis* coverage was higher after the fourth assessment day compared to the control treatment in the low treatment. This may be due to a delayed or prolonged development of *Borago officinalis* as a result of the herbicide treatment, which could be interpreted as hormesis or which could be due to a change in competition in the low treatment plot's community. These examples show that indirect effects and community effects (like competition or interception during application) in a plant community study can have an influence on single‐species endpoints and must be carefully considered. Furthermore, the examples demonstrate the influence of small changes in the study design: If the herbicide had been applied later or *Linum usitatissimum* had emerged prior to application, then the species would probably have shown a different response to the treatment. Application timing is always a crucial factor in NTTP field studies and has to be carefully chosen, as was also discussed for similar studies (Marrs et al. 1989; Nelemans et al. 2017). It seems reasonable to target vulnerable and sensitive growth stages of the test plants, for example, juvenile stages, as was done in the study presented here.

#### Community endpoints

The parameters measured and calculations made at the community level (total coverage, vegetation height, total shoot dry weight, diversity analysis, PRC) reflect the influence the herbicide treatment has on the plant community. The plant community includes the species from the soil seed bank that were not assessed at single‐species level. Because it is not possible to predict sensitivity of the soil seed bank species to the tested herbicide, their presence may change the measured effects at the community level. For the presented field study, the presence of the soil seed bank species will most likely result in lower sensitivity of the community compared to single‐species endpoints (except for results of PRC) because the sown species do not include monocotyledonous plants, which are not sensitive to the sulfonyl‐urea herbicide, but the soil seed bank species contain monocots. In addition, the ability of the plant community to compensate for reduced growth of 1 species by increased growth of another most likely also makes community‐level endpoints less sensitive. Indeed, when looking at the total coverage and total shoot dry weight, these measurements seem to show less effects than single‐species endpoints. Effects of the herbicide were only transiently detectable in the highest treatment, and no statistically significant differences between control, low, and medium treatment were observed. The transient nature of the detected effects has to be carefully discussed. Differences between controls and treatments may decline toward the end of the study, due not only to actual recovery but also to seasonal changes with reduced plant coverage at the end of the vegetation period. Nevertheless, other field studies reported recovery toward the end of the growing season (Marrs et al. 1989) and this may be the case in the study presented here as well.

Although there also was no significant difference for the species richness between treatments and control, evenness and Shannon‐Weaver Index both indicated differences for the medium and high treatment to the control (Figure 5). Again, these effects were transient as for the total plant coverage and total biomass. The diversity analyses showed highest sensitivity and may therefore be a suitable community‐level endpoint for evaluating effects of herbicides on nontarget plants. A prerequisite for using diversity analyses for this purpose is an appropriate choice of the right index to be used, based on the available data set. The distribution of data is crucial and must be carefully considered on a case‐by‐case level, especially for field studies where data distribution depend on external, unpredictable factors as, for example, the presence of soil seed bank species. Furthermore, the diversity analyses focus on the ecological point of view. Depending on the protection goal, it could be more meaningful to assess for the community (general) endpoints than to look at single‐species endpoints. If the ecosystem service to be protected would, for example, be climate regulation, it might be sufficient to evaluate diversity, abundance, or plain biomass. A recent study by Mkenda et al. (2019) lists ecosystem services that are provided by field margins. This list contains natural pest regulation, pollination, nutrient cycling, reduced offsite erosion, increased litter decomposition, protection of watercourses, et cetera. Further work will be needed to clarify how (community) endpoints from field studies could be used to support the ecosystem service approach and which type of endpoint (community, general, or species level) is necessary to ensure protection of the different types of ecosystem services.

Another method for analyzing and visualizing effects on the community level is the PRC, which is often used in other community‐level studies such as aquatic mesocosms (EFSA 2013). Principal response curve focuses on the differences in species composition in controls and treatments over time (Van den Brink and Ter Braak 1998, 1999) and could be used to assess the risk of tested herbicides to provisioning ecosystem services such as genetic resources or feed for specialized herbivorous animals, which rely on performance of a single species in a community. Because PRCs are based on data from a single species (coverage, in this case), they could be used to connect single‐species and community endpoints and answer questions related to both levels of analysis. In this specific study, the lowest NOER determined by the PRC is 0.12 g/ha a.s. This is also the lowest NOER determined by the single‐species assessments, for example, for coverage of *Borago officinalis*, *Phacelia tanacetifolia*, or *Centaurea cyanus* (Table 2). Accordingly based on our data, the use of the NOER derived of the PRC analysis in a risk assessment context can be regarded as protective for the single species. Further analysis is needed to estimate whether, in a test design such as the one proposed here, community‐level endpoints are always conservative enough to cover single‐species endpoints and subsequently to inform a protective risk assessment. If this is the case, this method of analysis could be used as a basis for an ecosystem‐services‐approach–based risk assessment for nontarget terrestrial plants that focuses on functional community endpoints instead of single‐species–level endpoints. An ecosystem service–based approach for the risk assessment, as is proposed in the *Scientific Opinion addressing the state of the science on risk assessment of plant protection products for non‐target terrestrial plants* (EFSA 2014), seems hard to follow when focusing only on single‐species endpoints.

### Comparing greenhouse and field study results

The standard greenhouse study reveals *Beta vulgaris*, *Glycine max*, and *Brassica napus* to be the most sensitive species among the plant species tested for effects of iofensulfuron‐sodium at this lower tier. These sensitive species were not included in the higher tier field study for practical reasons (e.g., crop plants will not grow well in a plant community; results of pilot studies, data not shown). The only species that was used in both study types was *Helianthus annuus*. Accordingly, comparing endpoints derived from the 2 study types is difficult. Nevertheless, it is a common approach to refine a lower tier risk assessment by using a higher tier endpoint based on testing of suitable but not identical species. For example, in aquatic mesocosm testing, rather than adapting the tested species in a community according to the most sensitive species from lower tier testing, instead one allows for an appropriate number of sensitive species in the test community. For a sulfonylurea herbicide, acting mostly on dicotyledonous plants, the used MEKA seed mixture certainly fulfills this requirement. Thus, a comparison of the relevant endpoints that could be used in a regulatory context is presented in the following part of the discussion. As discussed in the *Comparison of field study results to standard greenhouse study results* section, such a comparison could be based on (field vs greenhouse) NOERs only or on comparing greenhouse ER50s and field study NOERs. Both options are presented and discussed, and the overall conclusion remains the same for the herbicide under investigation. It has to be noted that this may not always be the case (e.g., when greenhouse ER50s are much higher than the corresponding greenhouse NOERs). The question, which endpoint is suitable for comparing study types and eventually refinement of the risk assessment, must be carefully considered for a future development of an NTTP field study guideline.

When comparing NOERs of the greenhouse study and field study, we see an increase in the lowest detected bound NOER of more than 10‐fold (0.01 g/ha a.s. for *Beta vulgaris* shoot dry weight in the greenhouse, 0.12 g/ha a.s. overall in the field). Fletcher et al. (1989) compared endpoint values for the same plant species tested in greenhouse and field studies for up to 17 herbicides and found response ratios between 0.52 and 3.26. For this comparison, a 95% probability was calculated that there will be less than a 2‐fold difference between greenhouse and field sensitivity. The same comparison can be done for *Helianthus annuus* for the data presented here (lowest NOER: ≥1.10 g/ha a.s. in the field and <0.12 g/ha a.s. in the greenhouse) and leads to a response ratio of 0.11 (greenhouse NOER divided by field NOER), indicating that based on NOERs, the current standard greenhouse studies may overestimate effects of herbicides on NTTPs. If the overall lowest greenhouse ER50 of 0.08 g/ha a.s. (*Beta vulgaris*, shoot dry weight) is compared to the field NOER of ≥1.10 g/ha a.s., the response ratio according to Fletcher et al. (1989) is 0.07, which further supports the assumption that greenhouse studies rather overestimate effects of herbicides. The currently used deterministic risk assessment for NTTPs uses a safety factor of 5 that is applied to ER50s resulting from greenhouse studies (EFSA 2002). Such a safety factor would not be necessary for the risk assessment based on results of a field study that is more realistic and represents a higher tier. For the assessment of the risk of iofensulfuron‐sodium toward NTTPs, applying the safety factor of 5 to the lower tier risk assessment would eventually lead to a higher level of protection (e.g., mitigation measures) as using the field NOER without an assessment factor (ER50 of 0.08 g/ha a.s. (*Beta vulgaris*, shoot dry weight): 5 = 0.016 g/ha a.s. vs field NOER of ≥1.10 g/ha a.s.). Accordingly, performing a cost‐ and time‐consuming field study would not be necessary to add protectiveness to the lower tier risk assessment.

## CONCLUSIONS

In the field study, community endpoints from the PRC were at the same level as single‐species endpoints and therefore could be a meaningful choice for a possible higher tier risk assessment for NTTPs. Further analysis is needed, to estimate whether community‐level endpoints are always at the same level as (or lower than) single‐species endpoints. If this is the case, they could be used as a basis for an ecosystem‐services‐approach–based risk assessment for nontarget terrestrial plants. Because the current NTTP risk assessment scheme generally stops at the level of a probabilistic tier 2 risk assessment based on ER50s, this scheme has to be expanded to incorporate NOERs derived from field studies. It has to be carefully evaluated whether the current risk assessment scheme could be adapted for use of results from the field study or whether a novel approach specifically designed for higher tier risk assessment may be necessary. It must be kept in mind that increased realism in the field also comes at a cost: Variability is increased, reproducibility is decreased, and interpretation of results is more difficult and has to be handled with care, as shown in the present study. Unintended growth of species from the soil seed bank is an unavoidable shortcoming of the proposed higher tier field study setup for assessing effects of herbicides on NTTPs, which must be managed as best as possible and be considered when evaluating results. An additional shortcoming is expected resource‐intense adaptations of the study design when testing different herbicides or testing in different regions. Overall, these shortcomings may limit the usefulness of the study results for conducting realistic and protective risk assessments. Comparison of the field study and standard greenhouse study results indicated that the current tier 2 risk assessment for NTTPs based on the standard greenhouse study results is protective in this case. Accordingly, at least for iofensulfuron‐sodium, conducting a labor‐ and cost‐intensive NTTP field study, which may also deliver variable and therefore less reliable results, would not add protectiveness. Whether the developed field study design and the assessed endpoints are suitable for a higher tier risk assessment of NTTPs in general depends on selection of the specific protection goal and requires further discussion. The presented study design may be considered as a possible NTTP reference tier.

## Disclaimer

This research has been conducted and funded by BAYER AG, Crop Science Division. Some authors are employed at Bayer AG or were paid for their work by Bayer AG. The sulfonyl‐urea herbicide iofelsulfuron‐sodium is developed but not sold by Bayer AG and not considered for a future registration.

## SUPPLEMENTAL DATA

The uploaded Supplemental Data file contains weather measurements for the duration of the field study as well as additional data for plant coverage and development of species from the MEKA mix, which were mentioned in the main document. In addition, phytotoxicity was measured for all 11 plant species from the MEKA mix, and detailed data on this parameter are shown in the Supplemental Data file.

## Supporting information

This article contains online‐only Supplemental Data.

Supporting information

## Data Availability

Bayer is making the science it owns available for review for the purpose of further scientific discovery, collaboration, and public awareness and of ensuring that robust science supports our technologies. The underlying research referenced in this article can be accessed by an email to the corresponding author Rena Isemer (rena.isemer@bayer.com).
