# Supplementary material for: A Field Study Method as a Potential Higher Tier Option to Refine Herbicide Risk Assessment for Nontarget Terrestrial Plants
Source: Integr Environ Assess Manag. 2020 Apr 16;16(5):691–705. doi: 10.1002/ieam.4263 (PMC7496580; doi:10.1002/ieam.4263)
Supplement: Supplementary file 1 — Supporting information [file IEAM-16-691-s001.docx]

**SUPPLEMENTAL MATERIAL**


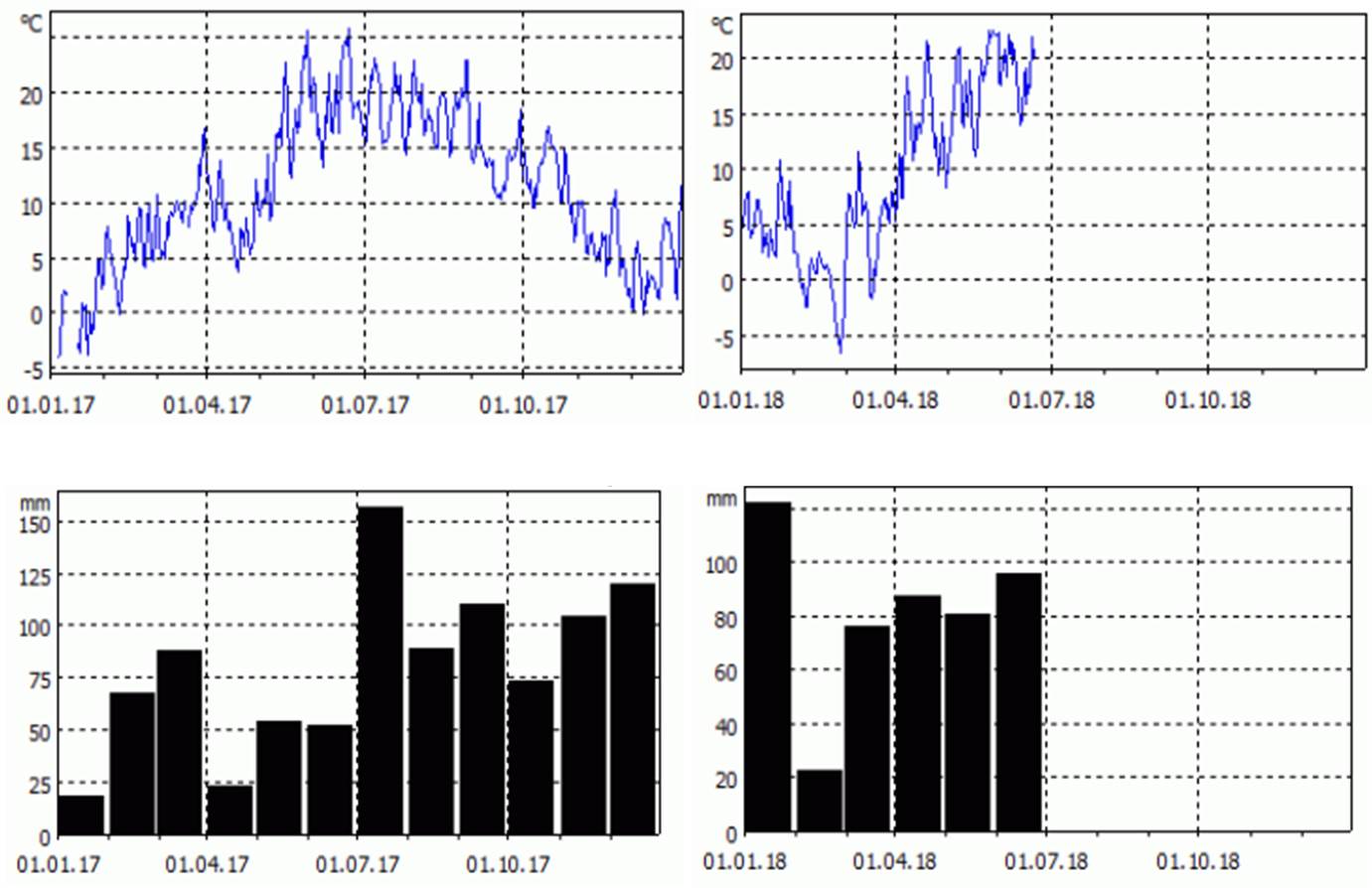
 Supplemental Figure 1: Daily mean temperature in °C (top) and monthly rainfall in mm (bottom) for the study site in Höfchen during the test period in 2017 (left) and 2018 (right).


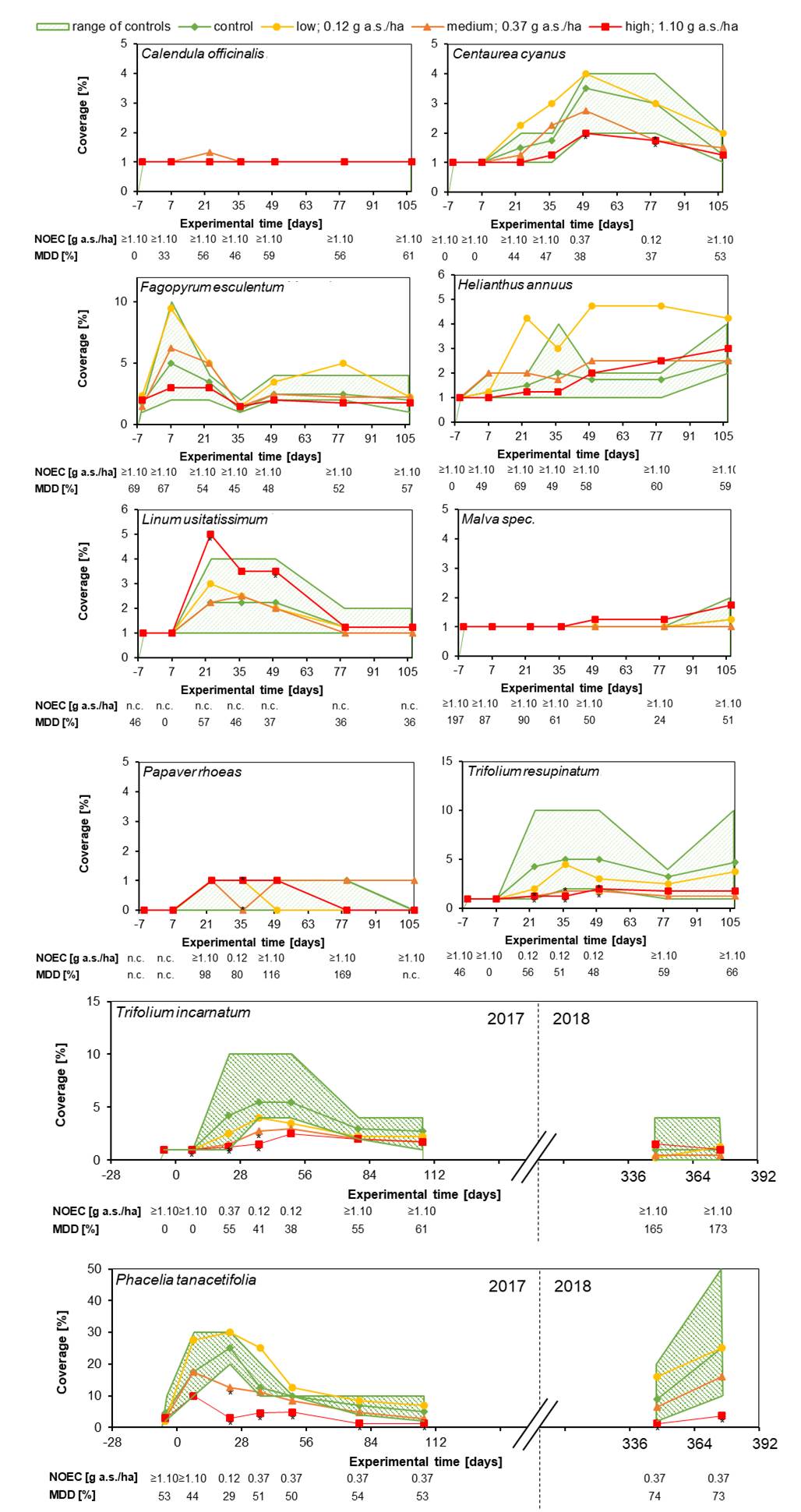


Supplemental Figure 2: Vegetation coverage of *Calendula officinalis*, *Centaurea cyanus*, *Fagopyrum esculentum*, *Helianthus annuus*, *Linum usitatissimum*, *Malva* spp*.*, *Papaver rhoeas*, *Trofilium resupinatum*, *Trifolium incarnatum* and *Phacelia tanacetifolia* (all species originating from the sown MEKA seed mixture). Average plant coverage with corresponding NOECs and MDDs for 2017 for the control and the three treatments groups at the respective assessment days is shown. Long term effects measured in 2018 could only be assessed for *Trifolium incarnatum* and *Phacelia tanacetifolia*, as the other species did not germinate or only in very few plots. *: significant differences according to William`s t-test with p-value <0.05; labeling of axis and captions are the same for all used graphs.


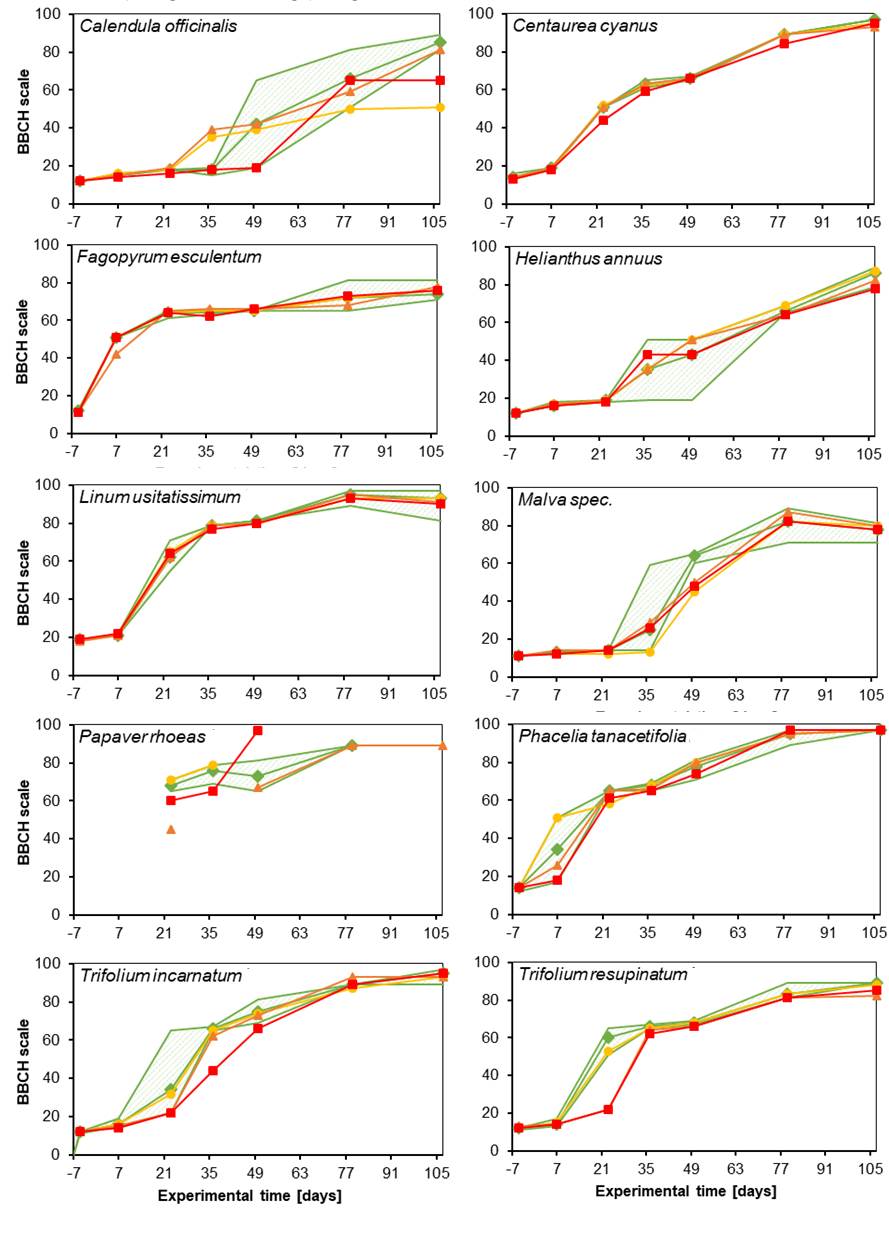


Supplemental Figure 3: Development of *Calendula officinalis*, *Centaurea cyanus*, *Fagopyrum esculentum*, *Helianthus annuus*, *Linum usitatissimum*, *Malva* spp*.* , *Papaver rhoeas*, *Trofilium resupinatum*, *Trifolium incarnatum* and *Phacelia tanacetifolia* (all species originating from the sown MEKA seed mixture). BBCH was not statistically evaluated.

Supplemental Table 1: Symptoms of phytotoxicity seen in *Borago officinalis*. The colouring scale from 0 to 20 reflects the range of observed phytotoxicity on the plots. Rating of phytotoxic symptoms: (0) no injury or effect; (A) slight symptom(s); (B) moderate symptom(s); (C) severe symptom(s); (D) total-plant symptom(s); (E) moribund. No phytotoxicity assessment was done before application of the test item (day -5).

Supplemental Table 2: Symptoms of phytotoxicity seen in *Calendula officinalis*. The colouring scale from 0 to 20 reflects the range of observed phytotoxicity on the plots. Rating of phytotoxic symptoms: (0) no injury or effect; (A) slight symptom(s); (B) moderate symptom(s); (C) severe symptom(s); (D) total-plant symptom(s); (E) moribund. No phytotoxicity assessment was done before application of the test item (day -5).

Supplemental Table 3: Symptoms of phytotoxicity seen in *Centaurea cyanus*. The colouring scale from 0 to 20 reflects the range of observed phytotoxicity on the plots. Rating of phytotoxic symptoms: (0) no injury or effect; (A) slight symptom(s); (B) moderate symptom(s); (C) severe symptom(s); (D) total-plant symptom(s); (E) moribund. No phytotoxicity assessment was done before application of the test item (day -5).

Supplemental Table 4: Symptoms of phytotoxicity seen in *Fagopyrum esculentum*. The colouring scale from 0 to 20 reflects the range of observed phytotoxicity on the plots. Rating of phytotoxic symptoms: (0) no injury or effect; (A) slight symptom(s); (B) moderate symptom(s); (C) severe symptom(s); (D) total-plant symptom(s); (E) moribund. No phytotoxicity assessment was done before application of the test item (day -5).

Supplemental Table 5: Symptoms of phytotoxicity seen in *Helianthus annuus*. The colouring scale from 0 to 20 reflects the range of observed phytotoxicity on the plots. Rating of phytotoxic symptoms: (0) no injury or effect; (A) slight symptom(s); (B) moderate symptom(s); (C) severe symptom(s); (D) total-plant symptom(s); (E) moribund. No phytotoxicity assessment was done before application of the test item (day -5).

Supplemental Table 6: Symptoms of phytotoxicity seen in *Linum usitatissimum*. The colouring scale from 0 to 20 reflects the range of observed phytotoxicity on the plots. Rating of phytotoxic symptoms: (0) no injury or effect; (A) slight symptom(s); (B) moderate symptom(s); (C) severe symptom(s); (D) total-plant symptom(s); (E) moribund. No phytotoxicity assessment was done before application of the test item (day -5).

Supplemental Table 7: Symptoms of phytotoxicity seen in *Malva* spp*.*. The colouring scale from 0 to 20 reflects the range of observed phytotoxicity on the plots. Rating of phytotoxic symptoms: (0) no injury or effect; (A) slight symptom(s); (B) moderate symptom(s); (C) severe symptom(s); (D) total-plant symptom(s); (E) moribund. No phytotoxicity assessment was done before application of the test item (day -5).

Supplemental Table 8: Symptoms of phytotoxicity seen in *Papaver rhoeas*. The colouring scale from 0 to 20 reflects the range of observed phytotoxicity on the plots. Rating of phytotoxic symptoms: (0) no injury or effect; (A) slight symptom(s); (B) moderate symptom(s); (C) severe symptom(s); (D) total-plant symptom(s); (E) moribund. No phytotoxicity assessment was done before application of the test item (day -5). As this species was not present in all plots at all times, white areas represent no assessment made.

Supplemental Table 9: Symptoms of phytotoxicity seen in *Phacelia tanacetifolia*. The colouring scale from 0 to 20 reflects the range of observed phytotoxicity on the plots. Rating of phytotoxic symptoms: (0) no injury or effect; (A) slight symptom(s); (B) moderate symptom(s); (C) severe symptom(s); (D) total-plant symptom(s); (E) moribund. No phytotoxicity assessment was done before application of the test item (day -5).

Supplemental Table 10: Symptoms of phytotoxicity seen in *Trifolium incarnatum*. The colouring scale from 0 to 20 reflects the range of observed phytotoxicity on the plots. Rating of phytotoxic symptoms: (0) no injury or effect; (A) slight symptom(s); (B) moderate symptom(s); (C) severe symptom(s); (D) total-plant symptom(s); (E) moribund. No phytotoxicity assessment was done before application of the test item (day -5).

Supplemental Table 11: Symptoms of phytotoxicity seen in *Trofilium resupinatum*. The colouring scale from 0 to 20 reflects the range of observed phytotoxicity on the plots. Rating of phytotoxic symptoms: (0) no injury or effect; (A) slight symptom(s); (B) moderate symptom(s); (C) severe symptom(s); (D) total-plant symptom(s); (E) moribund. No phytotoxicity assessment was done before application of the test item (day -5).

| **species** | **most sensitive measurement** | **MDD %** |  | **species** | **most sensitive measurement** | **MDD**  **%** |
| --- | --- | --- | --- | --- | --- | --- |
| *Beta vulgaris* | shoot dry weight | 13-18 |  | *Lycopersicon esculentum* | shoot dry weight | 12-13 |
| *Brassica napus* | shoot dry weight | 13-14 |  | *Allium cepa* | shoot dry weight | 10-11 |
| *Cucumis sativus* | shoot dry weight | 15-16 |  | *Lolium perenne* | shoot dry weight | 19-23 |
| *Glycine max* | shoot length | 8-9 |  | *Sorghum sudanese* | shoot dry weight | 27-36 |
| *Helianthus annuus* | shoot dry weight | 5 |  | *Zea mays* | shoot dry weight | 18-20 |

Supplemental Table 12: Minimum detectable differences (MDDs) for the greenhouse vegetative vigour study. MDDs are shown for the respective most sensitive measurement for each of the ten tested species and are displayed as minimum detectable difference to control in % of control as calculated by the ToxRat software. As MDDs are calculated separatedly for each of the six herbicide rates tested in the study, a range is shown.
